# Tunable growth of silver nanobelts on monolithic activated carbon with size-dependent plasmonic response

**DOI:** 10.1038/srep13587

**Published:** 2015-09-04

**Authors:** Hong Zhao, Yuesheng Ning, Binyuan Zhao, Fujun Yin, Cuiling Du, Fei Wang, Yijian Lai, Junwei Zheng, Shuan Li, Li Chen

**Affiliations:** 1 Department of Chemical Engineering, Jiangsu Marine Resources Development Research Institute, Huaihai Institute of Technology, Lianyungang 222005, P. R. China; 2 State Key Laboratory of Metal Matrix Composites, School of Materials Science and Engineering, Shanghai Jiao Tong University, Shanghai 200240, P. R. China; 3 College of Chemistry, Chemical Engineering and Materials Science, Soochow University, Suzhou 215123, P. R. China; 4 Green Support Materials Technologies (Shanghai) Co. Ltd. Shanghai 200240, P.R. China

## Abstract

Silver is one of the most important materials in plasmonics. Tuning the size of various silver nanostructures has been actively pursued in the last decade. However, silver nanobelt, a typical one-dimensional silver nanostructure, has not been systematically studied as to tuning its size for controllable plasmonic response. Here we show that silver nanobelts, with mean width ranging from 45 to 105 nm and thickness at *ca.* 13 nm, can grow abundantly on monolithic activated carbon (MAC) through a galvanic-cell reaction mechanism. The widths of silver nanobelts are positively correlated to the growth temperatures. The width/thickness ratio of the silver nanobelts can be adjusted so that their transversal plasmonic absorption peaks can nearly span the whole visible light band, which endows them with different colours. This work demonstrates the great versatility of a simple, green and conceptually novel approach in controlled synthesis of noble metal nanostructures.

The advancement of nanoscience and technology has enabled its wide applications in catalysis, biomedicine and plasmonics etc^1^. Over the past two decades, a wealth of methods has been developed to preparing nanostructures with controlled size and shape^2^,^3^,^4^,^5^. Among them, wet chemical synthesis of nanostructured noble metals or alloys^2^,^6^,^7^,^8^ consisting of Au, Ag, Pd and Pt has shown great capability and flexibility. Particular attention has been paid to Ag, since in the visible (vis) and near-infrared (NIR) bands, it can support surface plasmon polaritons with the highest plasmonic ability^9^. Surface plasma not only give rise to vivid colours characteristic of specific types of Ag nanostructures, but also drive increasing applications such as biological labelling, imaging, sensing, photothermal therapy and plasmonic circuitry etc. Various Ag plasmonic nanostructures in such forms as cubes^10^, octahedra^11^, plates^12^, rods and wires^13^,^14^ have been created, and sometimes their size can also be tuned to exhibit controllable plasmonic response. For example, by reducing the seed concentration in a seed-mediated micelle-directed synthesis, Ag nanorods with length/diameter aspect ratio ranging from 4.6 to 18 were prepared, which resulted in red-shift of the longitudinal plasmon mode from 885 nm to a wavelength longer than 1800 nm^13^. In addition, by collecting samples at different stages in a seed-mediated polyol synthesis, Ag nanocubes with different edge lengths ranging from 36 to 172 nm were obtained, which showed a gradual red-shift from 430 to 537 nm for the major dipole localized surface plasmon resonance (LSPR) peak^10^. The extinction spectra of Ag nanowires also demonstrated diameter-dependent red-shift from 391 to 547 nm for the transverse plasmon resonance, when the mean diameter was increased from 70 to 450 nm^15^. Notably, one-dimensional nanomaterial is of particular interest in that it supports propagating surface plasmon (PSP) and can be utilized as waveguides^16^. Beside nanowire, Ag nanobelt is another single-crystalline one-dimensional nanomaterial with a rectangular cross-section. Several methods (*see* Table 1) have been developed for the preparation of Ag nanobelts, such as reflux-induced nanoplates assembly^17^, polymer-controlled ascorbic acid reduction^18^, electrochemical method^19^,^20^, galvanic displacement^21^, and hydrothermal^22^ method etc. However, to our knowledge, there has been no report on the synthesis of Ag nanobelts with both tunable size and controlled plasmonic response. The scarcity in bulk production of Ag nanobelts with specified physical properties will certainly limit their application as nanoscale device components.

In our previous work^23^,^24^,^25^, a new method was developed to growing Ag micro/nano structures on monolithic activated carbon (MAC). Briefly, the functional groups on the interior surfaces, i.e., in the micropores of MAC (with sufficiently high BET area) caused the reduction of Ag^+^ ions on the exterior surface of MAC through a galvanic-cell mechanism. Abundant fluffy Ag products, like plant in a field, grew on the exterior surface of MAC and could be easily collected. This method differs from all other methods in that MAC acts as both solid substrate and reducing agent. The micropores of MAC do not confine the shape and size of Ag products, in contrast to commonly used substrates such as anodic aluminum oxide (AAO) containing cylindrical channels^20^,^26^. Rather, various structures such as dendrites^23^, belts, plates, flowers^25^ and cages^24^ can be selectively prepared by changing the identity and concentration of Ag precursors and modifying the MAC pretreatment. In particular, Ag belts can be routinely prepared in a large scale. However, the Ag belts obtained in our previous work actually had width of *ca.* 1 μm, and thickness of *ca.* 300 nm. This size is too large to have interesting optical and electronic effects^27^. In the current work, by forming metallic particles on MAC surfaces in prior as the growth initiator, and choosing sparingly soluble Ag_2_O as the Ag precursor to control Ag^+^ release and create a stable reaction environment, we prepared Ag nanobelts with width down to tens of nanometers, and thickness of *ca.* 13 nm in a high yield. All the Ag nanobelts have characteristic colours dependent on their cross sectional aspect ratio. Moreover, it was found that the temperature of the reaction media inherently affected the size of the Ag nanobelts, which in turn determined the optical absorption properties of the Ag nanobelts. In principle, we have been able to tune the size of Ag nanobelts for controllable plasmonic response.

## Results

**Figure 1:**
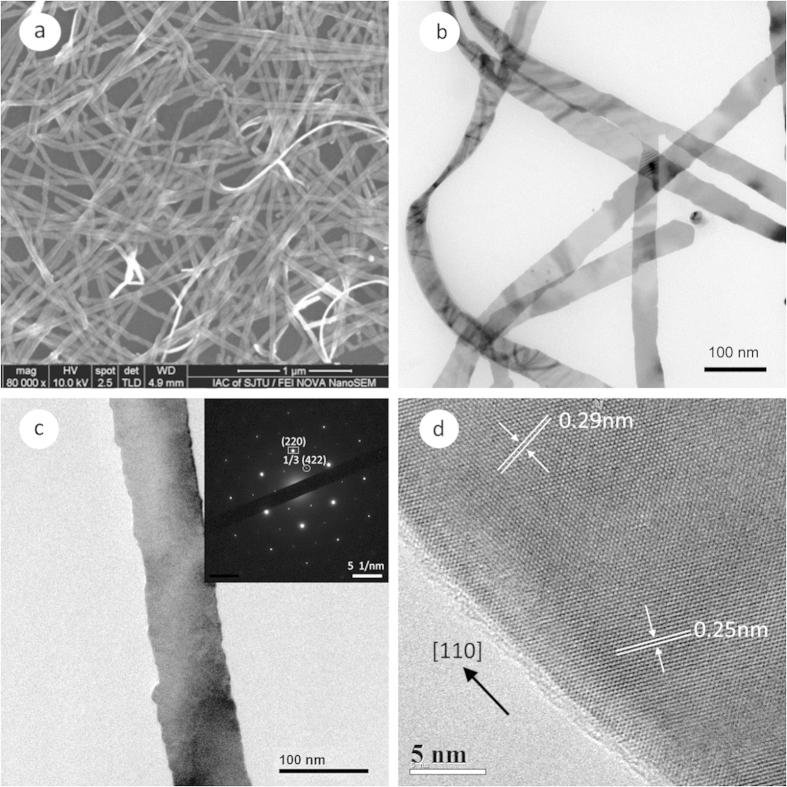
Typical EM images of silver nanobelts prepared on MAC. (**a**) SEM. Scale bar, 1 μm. (**b**) TEM. Scale bar, 100 nm. (**c**) TEM (Scale bar, 100 nm) with corresponding ED pattern as the inset (Scale bar, 5 1/nm). (**d**) HRTEM. Scale bar, 5 nm.

Figure 1 displays typical electron microscopic (EM) images of Ag nanobelts grown on MAC. The SEM image (Fig. 1a) shows that abundant wire-like products were obtained, with very few quasi-spherical particle impurities. Figure 1b is a typical TEM image of the Ag nanobelts. The mean width of the nanobelts shown in this image is *ca.* 45 nm. The thickness, as measured from a twisted nanobelt near the left side, is *ca.* 9 nm. This flat and smooth nanostructure lying on TEM grid is also confirmed by a Moire pattern at the right side, originated from the stacking of two or three nanobelts with different crystal orientation^17^. Figure 1c presents the TEM image of an individual nanobelt. Its selected area electron diffraction (SAED) pattern, as inserted in the upper right corner, has a six-fold symmetry. The *d* spacing of the planes corresponding to the brightest set of spots (squared) is calculated to be 0.144 nm, and the inner, weaker spots (circled) give a *d* spacing of 0.25 nm. This SAED pattern is identical to those reported previously^18^ and can be readily indexed to a single crystal fcc Ag in its 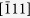 zone axis. The two diffraction spots, squared and circled, can be attributed to {220} and 1/3{422} reflections, respectively. The appearance of the formally forbidden 1/3{422} reflection spots also indicates that the nanobelts have flat top and bottom {111} facets^17^. In Fig. 1d, the HRTEM image of a Ag nanobelt exhibits clear fringes parallel to the edge with a spacing of 0.25 nm, which is due to the 1/3{422} reflection. A spacing of 0.29 nm for the planes perpendicular to the edge can be associated with {110} reflection, indicating that the Ag nanobelt is oriented along [110] direction. This [110] primary growth direction is also in accordance with most previous studies^17^,^18^,^22^. Occasionally, Ag nanobelt branches off into two stems at 60° (*see* Fig. 1b), and both are still along the <110> growth direction.

**Figure 2:**
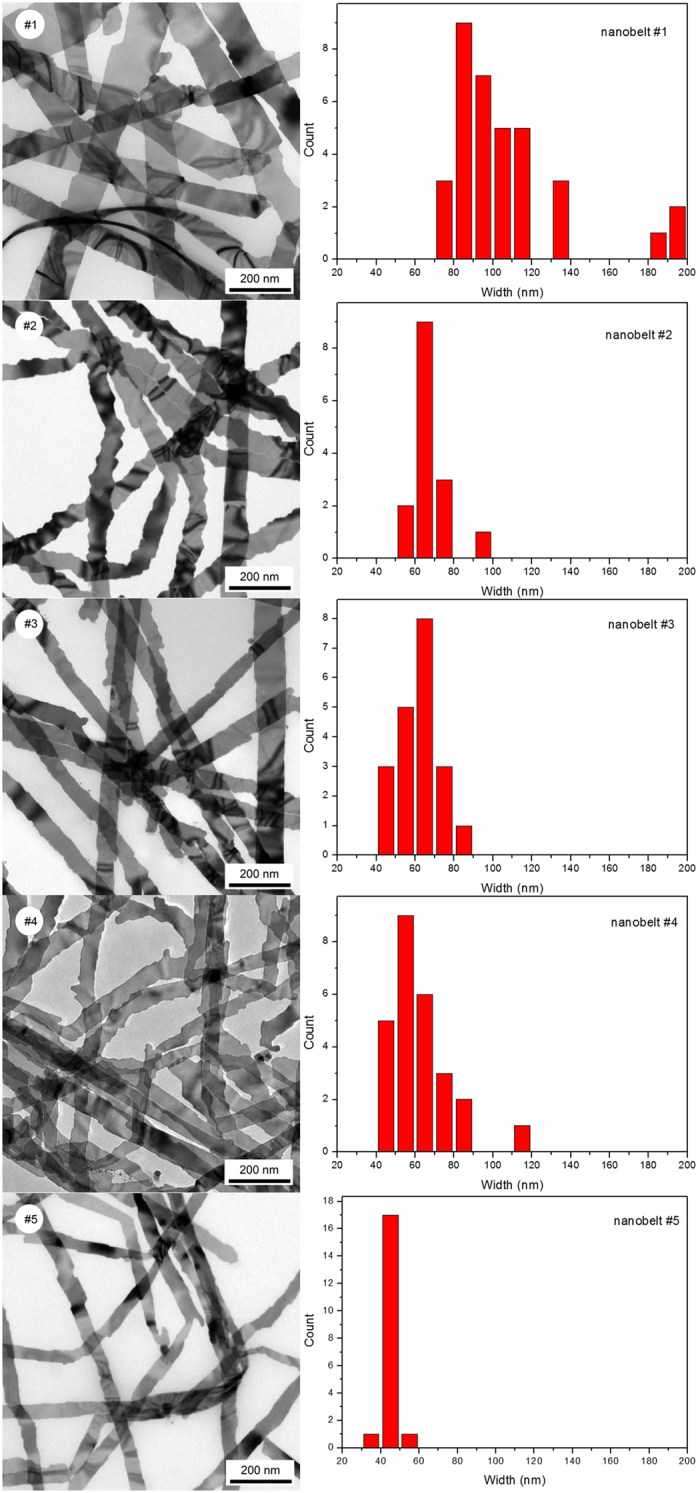
TEM images (left) and width histograms (right) of silver nanobelts prepared on MAC at different temperatures. Scale bars, 200 nm. (#1) 30 °C, (#2) 24 °C, (#3) 24 °C, (#4) 20 °C, (#5) 19 °C.

Figure 2 depicts the TEM images of five samples #1, #2, #3, #4 and #5, prepared at 30, 24, 24, 20 and 19 °C respectively, along with their width histogram. The mean widths of these nanobelts are listed in Table 2. They are 105.4 nm (#1), 67.5 nm (#2), 62.4 nm (#3), 61.8 nm (#4) and 45.3 nm (#5). These results suggest that the width of Ag nanobelts decreases at lower growth temperature. However, beside temperature, other experimental parameters such as MAC structure and properties, and Ag seeds loading condition etc. may have a profound effect on the size of Ag nanobelts. These experimental conditions had not been able to strictly controlled, and they often smeared the width-temperature relationship. Upon hundreds of experimental tests, it turned out to be a general trend that the width of Ag nanobelts positively correlated to the reaction temperature.

Due to the scarcity of nanobelts with their side edge parallel to the electron beam, it is difficult to get mean thickness of Ag nanobelts using TEM. Atomic force microscope (AFM) was employed to measure the thickness for the five samples mentioned above. A typical AFM image of an individual nanobelt is shown in Supplementary Fig. S1, and the section analysis yielded thickness of 13.607 nm (shown as “vertical distance” in the image). For the five samples mentioned above, the thicknesses of *ca.* 10 nanobelts per sample were measured, and their mean thicknesses are also listed in Table 2. These nanobelts have mean thickness between 11 and 16 nm. Apparently, there is no simple relationship between the reaction temperature and mean thickness, suggesting that other parameters may affect the thickness no less than reaction temperature.

**Figure 3:**
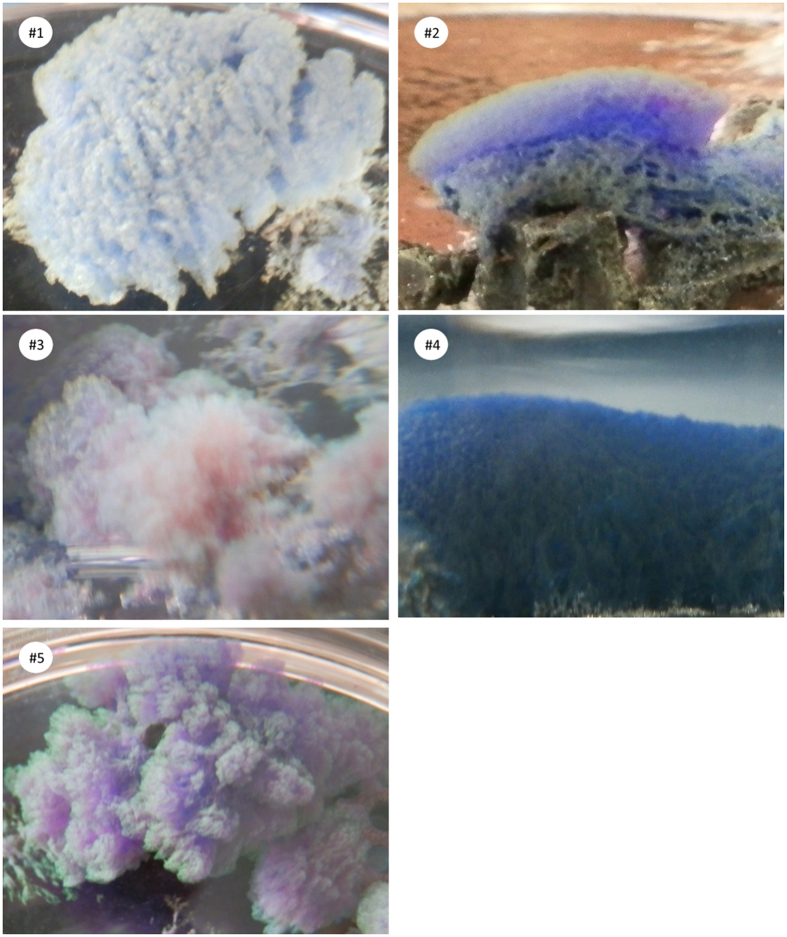
Digital photos of silver nanobelts growing on MAC at different temperatures. (#1) 30 °C, (#2) 24 °C, (#3) 24 °C, (#4) 20 °C, (#5) 19 °C. *Note* for sample #2, black MAC can be found under blue-purple nanobelts.

Importantly, these Ag nanobelts prepared on MAC showed different colours under common daylight illumination. For the five Ag nanobelt samples mentioned above, digital photographs were taken and displayed in Fig. 3. Nanobelts #1, #2, #3, #4 and #5 appear light-blue, blue-purple, red, blue, and purple respectively. These as-grown colourful nanobelts resting on the MAC substrate (as most clearly seen in #2 of Fig. 3), looking like colourful seaweeds, can be easily detached by plastic tweezers and dispersed in suitable solvents. Notably, although there have been a variety of methods to preparing noble metal nanobelts^28^, and some methods seem to have some tunability on their size^20^,^22^, to our knowledge, there has been no report on the synthesis of noble metal nanobelts with different colours. Therefore, our simple synthetic method could provide a competitive alternative to tune the size and physical properties of noble metal nanobelts.

**Figure 4:**
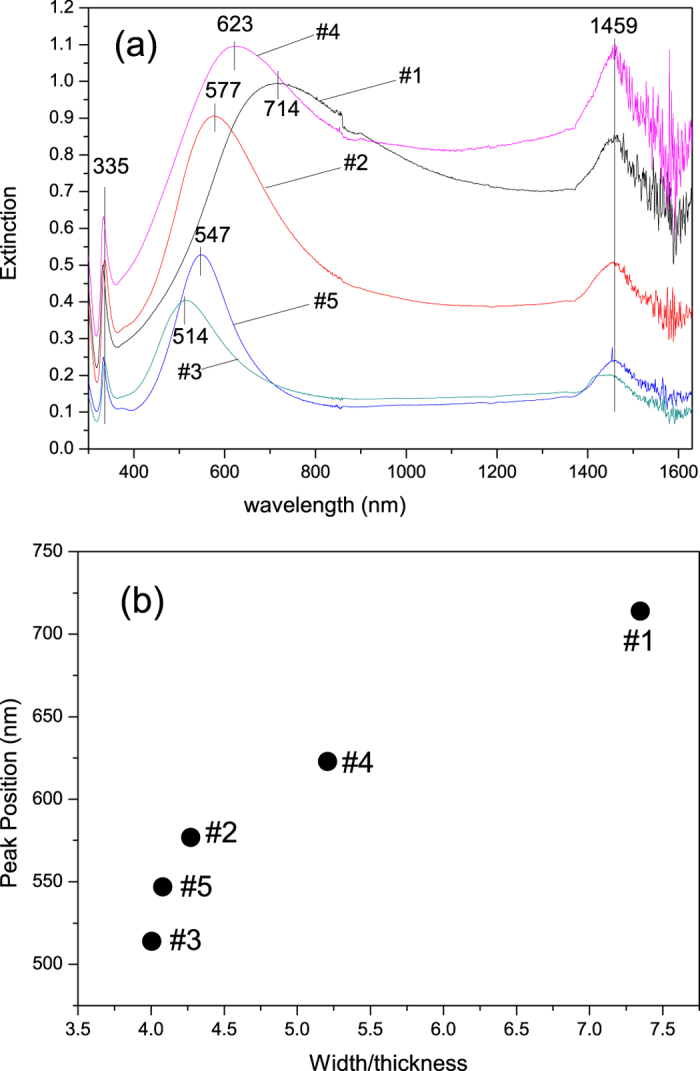
Extinction spectra and their dependence on the size of silver nanobelts. (**a**) Extinction spectra of silver nanobelts. (**b**) The dependence of the visible light absorption peak wavelength on the size (width/thickness ratio) of silver nanobelt.

It is well known that the brilliant colour of noble metal nanoparticles is resulted from absorption and scattering of light associated with surface plasmon resonance (SPR)^9^, i.e. the collective oscillation of free electrons in phase with the alternating electric field of the incident light. Using UV-Vis-NIR to measure the extinction spectra is a traditional and most prevalent way to characterize the plasmonic response of noble metal nanoparticles. The extinction spectra of the five nanobelt samples mentioned above are illustrated in Fig. 4a. Each happens to exhibit three distinctive peaks at the UV, visible and IR band respectively. While the wavelength of the UV (335 nm) and IR (1459 nm) peaks are relatively invariant, that of the visible peak shifts significantly from 514 nm to 714 nm. (Yellow Ag nanobelts with their visible peak wavelength at 474 nm were also obtained in our experiments at reaction temperature 13 °C. However, due to the lack of structural characterization they are not included here. *see* Supplementary Fig. S2). Notably, though there have been many experimental and theoretical studies concerning UV-Vis-NIR extinction spectra of other nanostructures, those for Au and Ag nanobelts are quite scarce^17^,^22^,^29^. Here, the UV peak at 335 nm may be tentatively attributed to the out-of-plane quadrupole plasmon resonance mode, based on discrete dipole approximation (DDA) calculations by Schatz *et al.*^17^,^30^. The invariance of this peak is similar to the quadrupole resonance of Ag nanowires, whose wavelength is also independent on their diameter^15^. The NIR peak at 1459 nm should be ascribed to the first overtone of O-H stretch of the ethanol solvent^31^,^32^.

The change of visible peak wavelength in the extinction spectra (Fig. 4a) corresponds directly to the colour of these Ag nanobelts (Fig. 3), i.e., samples that appear blue have longer extinction wavelengths, while those showing red tint display shorter ones. Recently, J. H. Hafner *et al.*^29^ used dark field polarized light microscope to collect the extinction spectrum of individual Au nanobelt. By correlating the peak wavelength to the width/thickness ratio, assisted by finite-difference time domain (FDTD) calculations, they were able to establish positive relationship between a transversal antisymmetric plasmon peak wavelength and the width/thickness ratio. Inspired by their work, we also included in Table 2 the size (mean width as measured by TEM, and mean thickness as measured by AFM) and visible peak wavelength for the five nanobelt samples. The relationship between the visible peak wavelength and the width/thickness ratio was plotted in Fig. 4b. Clearly, our results agree well with the positive correlation as found by Hafner *et al.*^29^. Notably, both their single and our collective spectral peak wavelenths somewhat deviate from the approximately linear relationship with width/thickness ratio proposed theoretically^29^. Therefore, the visible peaks observed in our extinction spectra can be assuredly attributed to the transversal plasmon mode. The blue- or red-shift of this peak as resulted from the variation of the width/thickness ratio, which is in turn tunable by experimental condition such as the reaction temperature, accounts for the different colours of Ag nanobelts prepared on MAC. This size-colour relationship was also confirmed by our XRD results, as presented in Supplementary Fig. S3.

## Discussion

In this work, for the first time, we provide a method to growing Ag nanobelts with size-dependent plasmonic response in the visible wavelengths. Among all the available approaches for the synthesis of Ag nanobelts (Table 1), the hydrothermal^22^ and electrochemical^20^ methods also showed some tunability on the size, including the width and thickness. However, the reported data showed that the nanobelt sizes were a bit too large to give characteristic plasmonic absorption in the visible light region. Both reports^20^,^22^ gave nanobelts’ dimension somewhat larger than the electron mean free path (EMFP) of 52 nm for Ag^33^. In fact, only a broad peak centred at 392 nm was observed in the extinction spectrum by Yang^22^, which was attributed to the transverse plasmon absorbance, and no extinction spectra were given by Liu^20^. On the other hand, in our current work, we prepared Ag nanobelts with their mean thickness and width at 11 ~ 16 and 45 ~ 105 nm respectively. According to Murphy *et al.*^14^, interesting optical and electronic effects are expected on the 10 ~ 100 nm scale for metallic nanoparticles. Therefore, the sizes of Ag nanobelt prepared in this work fall within the active scale for plasmon excitation, which explains why they exhibit excellent plasmonic response.

**Figure 5:**
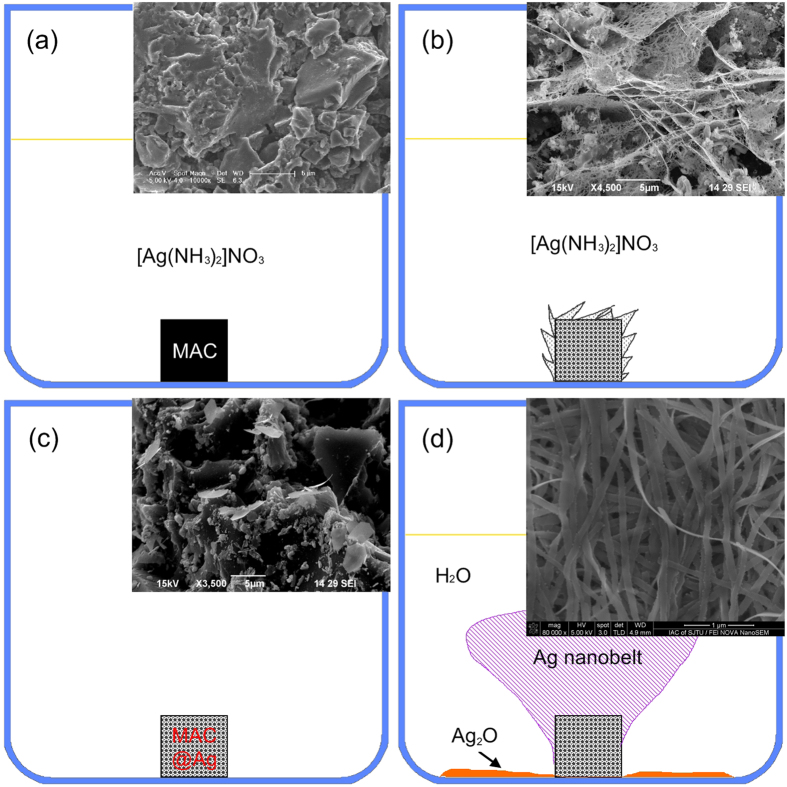
A schematic illustrating the synthetic procedure of silver nanobelts on MAC. (**a**) Firstly, an MAC was immersed into a freshly prepared [Ag(NH_3_)_2_NO_3_] solution. (**b**) Secondly, after 24 hr, silver micro- belts/plates were prepared on the surface of MAC. (**c**) Thirdly, the MAC in (**b**) was stripped of loosely attached silver, ultrasonicated in ethanol and dried in air. It was labelled as MAC@Ag after the treatment. (**d**) Lastly, after immersing MAC@Ag in DI water containing Ag_2_O powder for 48 hr, fluffy silver nanobelts grew on MAC@Ag substrates. The insets show SEM images of (**a**) untreated MAC, (**b**) silver micro belts/plates grown on MAC, (**c**) the exterior surface of MAC@Ag and (**d**) silver nanobelts.

**Figure 6:**
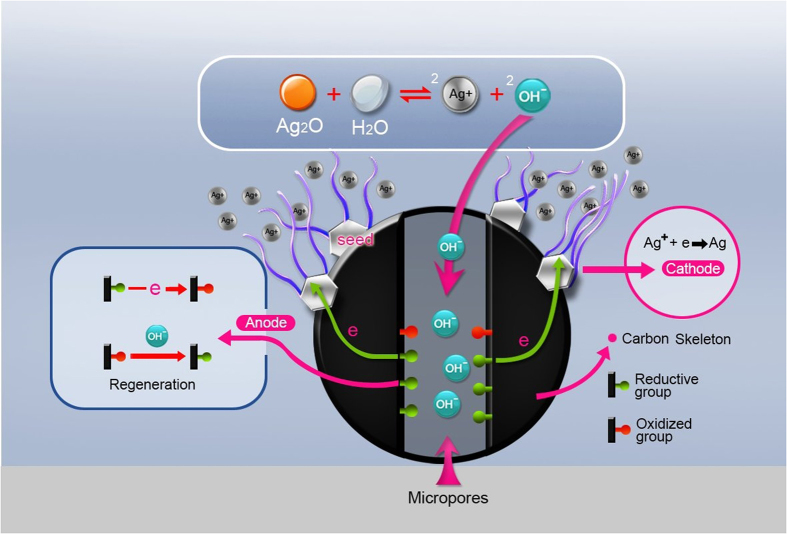
A Schematic of the galvanic-cell mechanism for the growth of silver nanobelts by immersing monolithic activated carbon (MAC), preloaded with metal seeds, in sparingly soluble Ag_2_O supernatant.

It is imperative to understand why the nanobelt size can be greatly reduced from micrometer^25^ to nanometer scale. Figure 5 displays the synthestic procedure of Ag nanobelts on MAC (*see* Methods for more detail) in this work. Briefly speaking, colourful Ag nanobelts (in Fig. 5d) were prepared by immersing MAC preloaded with metal particles (MAC@Ag as shown in Fig. 5c) in water, where sparingly soluble Ag_2_O powders had been placed at the bottom of the beaker. Fluffy Ag nanobelts grew on the exterior surface of MAC@Ag as resulted from continuous reduction of Ag^+^ (dissolved from Ag_2_O) by reductive functional groups (such as –OH or –CH=O) on the interior surface of MAC@Ag through a galvanic cell reaction mechanism (Fig. 6)^23^,^24^,^25^. There are mainly two differences from our previous work^25^, where Ag belts only at the micrometer scale were produced. Firstly, MAC should be preloaded with tightly bound metal particles. Beside Ag, we have found that preloaded Au, Pt or Pd particles can also be used to initiate the growth of nanometer-scaled Ag belts. There is no specific requirement for the size and shape of the preloaded metal particles. In our experiments, the MAC that had been used to grow Ag micro-belts or plates in [Ag(NH_3_)_2_]NO_3_ aqueous solution^25^ has proven to be excellent candidates only if the loosely-attached micro-belts or plates were removed and the MAC ultrasonicated and dried (*see* Fig. 5a–c). Secondly, instead of [Ag(NH_3_)_2_]NO_3_, Ag_2_O was used as the Ag precursor in our current work. We have found that, at appropriate concentrations, say, 10^−4^ M, [Ag(NH_3_)_2_]NO_3_ can also be occasionally used to grow nanometer-scaled Ag belts, but the reproducibility cannot stand test. On the other hand, Ag_2_O is a robust precursor for growing Ag nanobelts.

The metal particles preloaded on MAC may act as both electron conductors and heterogeneous nucleation sites. Since Ag^+^ ions were reduced by functional groups inside MAC micropores through a galvanic-cell mechanism, i.e., electrons transfer from inside MAC to the exterior surface during nanobelt growth, the tightly bound metal particles should be better electron conductors. Thus Ag^+^ ions should be preferentially reduced to Ag^0^ on these preloaded metal particles rather than on the carbon substrate. In addition, it may be due to the catalytic properties of metal particle surfaces^34^ that Ag^+^ ions, at so low concentrations as provided by Ag_2_O dissolution in DI water, could be reduced, overcome the nucleation barrier and grow into nanobelts. Without preloaded noble metal particles, we have found that no Ag product would appear after a prolonged immersion of MAC into a beaker containing Ag_2_O and DI water. This observation further implies the prerequisite of metal particles as the growth initiator of Ag nanobelts. These metal particles also provide substrates, breaking the symmetry of the reaction environment, and imposing a geometric constraint appropriate for anisotropic growth of silver nanobelts^35^,^36^. Further, we have found that Ag nanobelts at thickness of *ca.* 13 nm could abundantly grow on MAC preloaded with micrometer-scaled metal particles (*see* the inset of Fig. 5c for a typical SEM image of MAC@Ag surface). To explain this phenomenon, it is suggested that these Ag nanobelts probably grow out from sharp edges or vertices of the metal particles^21^. Since the growth of Ag nanobelts is a diffusion limited kinetically controlled process^2^, the corners of the metal particles may have much more opportunity to interact with Ag^+^ ions and become sites of rapid growth^37^, ensuring the small size of the nanobelts irrespective of the initiating metal particles.

Choosing Ag_2_O as Ag precursor is another important measure toward successful synthesis of Ag belts at the nanometer scale. The solubility of Ag_2_O in water at 20 °C is 0.0013 g/100 mL^38^, which is equivalent to 0.112 mM Ag^+^. This concentration is lower than typical experimental conditions for wet chemical synthesis of Ag nanoparticles. For example, Bai *et al.*^18^ synthesized Ag nanobelts by reducing 0.5 mM AgNO_3_ with ascorbic acid. Cao *et al.*^39^, though also used saturated Ag_2_O supernatant solution (*ca.* 0.2 mM Ag^+^) as Ag precursor, applied a strong reducing agent, hydrazine hydrate and produced triangular and hexagonal nanoplates, with edge length varying from 40 to 145 nm, and thickness at *ca.* 10 nm. In fact, in our previous work^25^, when 0.1 mM [Ag(NH_3_)_2_]NO_3_ was used as the precursor, the main product was also hexagonal plates (but having much larger sizes, with edge length at several μm and thickness at *ca.* 200 nm). In our current work, at first glance, the low Ag^+^ concentration, coupled to only modest reductive functional groups (such as –OH and –CH=O covalently bound to MAC)^25^, may not be a favourable condition for crystal nucleation, according to LaMer’s nucleation mechanism^40^. However, it may be due to the large BET area and high ion absorption properties of MAC, that Ag^+^ can be enriched near the exterior surface of MAC and reduced to Ag^0^ on the catalytic metal particles, creating a local layer where heterogeneous nucleation may occur. At the growth stage, the solid Ag_2_O may serve as a non-exhaustible reservoir for continuous supply of Ag^+^ onto the nanobelts growth frontier, creating a stable reaction environment. Notably, a scrutinized comparison of the nanobelts prepared in this work and the nanoplates synthesized by Cao *et al.*^39^ revealed a hidden similarity. Both use Ag_2_O as Ag precursor and both have thickness at 5 ~ 20 nm and edge length (or width) varying from 40 nm to over 100 nm. This similarity suggested that the low solubility of Ag_2_O played a key role in reducing the size of Ag particles down to nanometer scale.

Although size control of many nanostructures such as nanowires^15^, nanorods^13^, nanocubes^10^, nanoprisms^12^,^39^ has been reported by many researchers, surprisingly, there have been very few papers dealing with cross-sectional size control of wet-chemically synthesized nanobelts. In hydrothermal synthesis of Ag nanobelts, Yang *et al.*^22^ increased the [citrate^3−^]/[Ag^+^] ratio from 1:1 to 20:1, while keeping [Ag^+^] constant, and found that the nanobelt width decreased gradually from 212 nm to 70 nm. Here the citrate served both as reducing and capping agent. They attributed the size-reduction to the improved protecting ability exerted by citrate^3−^. Liu *et al.*^20^, in their electrochemical growth of Ag nanobelts, found that by improving [NH_3_·H_2_O] in the electrolyte solution, the nanobelt thickness increased from 7 to 98 nm. The increase of the thickness of Ag nanobelts was ascribed to the reduction of the thickness of double layer on the surface of alumina nanochannels, associated with higher electrolyte (NH_4_^+^) concentration. In our current work, the reaction beaker contained only MAC@Ag and saturated Ag_2_O in DI water. It was quite “clean”^41^ without any water soluble organic or inorganic reagent. Therefore, reaction temperature was the only adjustable parameter during the growth of Ag nanobelts. Our results indicated that the reaction temperature positively correlated with nanobelt width (Fig. 2). To our knowledge, there has been no study describing the effect of reaction temperature on the size of wet chemically synthesized nanobelts. In their vapour-solid (VS) deposition for the growth of α-Al_2_O_3_ nanobelts, Fang *et al.* also found a positive correlation between temperature and nanobelt width within four deposition zones from 1100 to 1300 °C^42^. Higher partial pressure and higher flow rate of the reactant associated with higher temperature were credited to the larger cross-section. In the gallium-catalysed thermal oxidation of iron substrate for the growth of α-Fe_2_O_3_ nanostructure, Yang *et al.*^43^ found the width of α-Fe_2_O_3_ nanobelt increased with temperature at 600 ~ 750 °C. The increase of width was attributed to higher supersaturation and higher diffusion rate of Fe and O in Fe-Ga nanoscale droplet, which favoured the two-dimensional nucleation with larger width. In our current work, firstly, the solubility of Ag_2_O increases from 0.0013 to 0.0053 g/100 ml if the water temperature is elevated from 20 to 80 °C^38^. Secondly, the calculated mobility of Ag^+^ in aqueous solution can increase from 5.15 × 10^−4^ to 7.04 × 10^−4^ cm/s as the temperature is elevated from 15 to 30 °C^44^. As the solution was kept stationary during our experiment, both these factors mean that at higher temperatures, more Ag^+^ can be supplied to nanobelt growth frontier. Therefore, in principle, the width of Ag nanobelts can be increased with the enhanced supply of Ag^+^ at higher temperatures, similar to α-Al_2_O_3_ nanobelts prepared by vapour-solid deposition^42^.

As to the thickness of Ag nanobelts, although some data in Table 2 (such as nanobelts #2, #3, #4 and #5) suggest that it also has positive correlation with the growth temperature, this trend cannot be verified as the thickness of nanobelt #1 deviates significantly. We presume that the thickness of Ag nanobelt was more dictated by the metal particles preloaded on MAC, which, however, had not been controlled in this work. In their catalyst-assisted vapour-liquid-solid (VLS) growth of Ga_2_O_3_ nanobelts^45^, Zhang *et al.* also found that the thickness of Ga_2_O_3_ nanobelt was similar to the size of Sn catalyst, while the width had no relationship with the Sn particle size. Notably, as typified by some “zigzag” nanobelts shown for sample #2 (Fig. 2), the width of many Ag nanobelts prepared in our work often changes along the growth direction. In fact, previous reports frequently show images of individual nanobelt with constant thickness and varying width along the growth direction^46^,^47^,^48^. It is implied that for most single-crystalline nanobelts, the thickness can be determined in the early nucleation stage and keeps constant, while the width is more susceptible to the environment such as the fluctuation of the precursor concentration and the diffusivity etc. during crystal growth. However, this rule remains to be tested. Additionally, the roughness of silver nanobelts prepared in this work may induce scattering loss and affect the propagation of surface plasmon resonance (SPR) along the belts. Possible solution to reduce the roughness may include a special experimental design to reduce the fluctuation of [Ag^+^], which would be investigated in future work.

## Conclusion

In summary, a method was developed to growing Ag nanobelts on monolithic activated carbon (MAC) with tunable size and size-dependent plasmonic response. Ag nanobelts were synthesized by reducing Ag^+^ with surface functional groups on MAC through a galvanic-cell mechanism. Loading metal particles on MAC as heterogeneous nucleation sites, and choosing sparingly soluble Ag_2_O as Ag precursor to control the release of Ag^+^ cooperatively facilitated stable growth of fluffy Ag nanobelts with width at tens of nanometers and thickness down to *ca.* 13 nm. The width of Ag nanobelts was found positively correlated to the reaction temperature, while the thickness may be more dictated by the metal particles preloaded on MAC. The width/thickness ratio also positively correlated to the transversal plasmonic absorption peak wavelength, which was located at the visible light region and endowed nanobelts with tunable colours. This work represents the first attempt in chemically producing Ag nanobelts with tunable size and size-dependant plasmonic response. The controllable plasmonic response is very promising for novel applications such as nanomedicine, sensing, high confinement plasmonic waveguides, and three-dimensional plasmonic nanocomposites^49^ etc.

## Methods

### Materials

Monolithic activated carbon (MAC) samples, derived from corn straw, were provided by Qufu Tembton Carbon Technology LLC. Shandong, China. Their compositional and structural information was similar to that described in our previous work^25^. In brief, they had a BET surface area of *ca.* 500 m^2^/g, and apparent density of *ca.* 0.8 g/cm^3^. As measured by X-ray photoelectron spectrometer, they had 85 atom% of C, ~10 atom% of O, and minor impurities such as Zn, Si, and S etc. Ag_2_O, AgNO_3_, ammonia water (25 ~ 28 wt%), and anhydrous ethanol were purchased from Shanghai Chemical Reagent Co. Ltd. Deionized (DI) water was generated from a TTL-30C Ultrapure Water Generator with electrical resistivity of 18.2 MΩ·cm. All chemicals were of analytical grade and used without further purification.

### Synthesis of silver nanobelts

#### Preparation of [Ag(NH_3_)_2_]NO_3_ solution

[Ag(NH_3_)_2_]NO_3_ solution was prepared by continuously dropping ammonia water into 1 M AgNO_3_ solution until the dissolution of brown Ag_2_O precipitate. Then the 1 M [Ag(NH_3_)_2_]NO_3_ solution was diluted to 10^−4^ M with deionized (DI) water, which was immediately used for the preparation of MAC loaded with Ag seeds (MAC@Ag).

#### Preparation of MAC with silver seeds (MAC@Ag)

MAC@Ag was prepared according to a procedure to preparing Ag hexagonal plates on MAC as described previously^25^. Firstly, the as-received MAC samples were cut into small pieces (*ca.* 0.2 g) and ultrasonicated in DI water to eliminate impurities. Then they were immersed into freshly prepared [Ag(NH_3_)_2_]NO_3_ solution (10^−4^ M) for 24 hr to grow Ag plates. Consequently, MAC was taken out and ultrasonicated in anhydrous ethanol for 1 hr to remove loosely attached Ag product, and finally dried at 60 °C for 12 hr. The obtained MAC loaded with tightly bound Ag particles was labelled as MAC@Ag.

#### Synthesis of silver nanobelts

Firstly, *ca.* 0.2 g Ag_2_O powder was added into a glass beaker containing 250 ml DI water to prepare saturated AgOH solution (with most Ag_2_O stay in the bottom of the beaker since Ag_2_O is only sparingly soluble in water). Then, MAC@Ag was immersed into AgOH solution for 48 hr to synthesize Ag nanobelts. Temperature of the reaction medium was controlled by placing the glass beakers in a water bath. All experiments were carried out in the dark to avoid Ag photoreduction. The colourful Ag nanobelt products, as shown in Fig. 3, were detached from MAC@Ag substrate with tweezers, purified with cycles of cleansing and centrifuging by DI water or ethanol, and finally dispersed in anhydrous ethanol for sample characterization.

### Characterization

#### Photograph

All photographs in this work were taken with a digital camera (OLYMPUS SZ-10).

#### SEM

Scanning electron microscopic (SEM) images were collected on an FEI NOVA NanoSEM 230 instrument with a TLD detector. The accelerating voltage, working distance was set at 10 kV, 5 mm, respectively. SEM specimen was prepared by drying droplets of Ag nanobelts suspension in ethanol on silicon wafers.

#### TEM

Transmission electron microscopy (TEM) and selected-area electron diffraction (SAED) experiments were performed on a Philips CM10 instrument at an accelerating voltage of 160 kV. TEM specimen was prepared by drying droplets of Ag nanobelts suspension in ethanol on a carbon-film coated copper grid. TEM was mainly used to statistically measure the width of Ag nanobelts. High resolution electron microscopy (HREM) experiments were performed on a JEM 2100F at an accelerating voltage of 200 kV.

#### AFM

The thickness of *ca.* 10 nanobelts was measured for each sample by atomic force microscopy (AFM). A multimode Nanoscope IIIa scanning probe microscope (Digital Instruments) mounted with Si tips with tip radius less than 10 nm and force constant at *ca.* 40 N/m was operated in a tapping mode. The resonant frequency and scanning rate were set at *ca.* 300 kHz and 1 Hz respectively. AFM specimen was the same as that used for TEM experiments, i.e. droplets of Ag nanobelts suspended in ethanol were dried on the carbon-coated copper grid.

#### Extinction spectra

Extinction spectra of Ag nanobelts suspended in ethanol were recorded at 25 °C using a PerkinElmer Lambda 750S UV-Vis-NIR spectrophotometer with a 1-cm quartz cuvette.

#### XRD

The powder X-ray diffraction (XRD) pattern of Ag nanobelts (and other Ag products) was obtained by using a Bruker AXS D8 X-ray diffractometer with Cu Kα radiation (λ = 1.5418 Å). The 2θ was scanned from 10° to 90° at 2°/min with 0.02° sampling frequency. The tube voltage, current was set at 30 kV, 40 mA respectively. XRD samples were prepared by covering one side of a 3 × 2 × 0.2 cm^3^ silicon chip with Ag nanobelt powders.

## Additional Information

**How to cite this article**: Zhao, H. *et al.* Tunable growth of silver nanobelts on monolithic activated carbon with size-dependent plasmonic response. *Sci. Rep.* **5**, 13587; doi: 10.1038/srep13587 (2015).

## Supplementary Material

Supplementary Information

## Figures and Tables

**Table 1:** Comparison of main synthetic methods of silver nanobelts on their size tunability and plasmonic response.

Method	Width (nm)	Thickness (nm)	Main plasmon resonance	Size tunable?	Additional Remarks
Reflux-induced assembly^17^	7–30	N.A.	N.A.	N.A.	Low yield (5%)
Polymer-directed reduction^18^	60–100	30–40	N.A.	N.A.	Yield of nanobelts sensitive to the amount of polymer
Electrochemical^19^	80–150	*ca.* 20	Main 380 nm, shoulder 410 nm	N.A.	Nanobelts ordered and ultralong (5 mm)
Electrochemical^20^	>200	7 ~ 98, tunable	N.A.	Thickness (by [NH_3_·H_2_O])	Product morphology sensitive to reduction potentials
Galvanic displacement^21^	>200	N.A.	N.A.	N.A.	Both the surfaces and edges of nanoblets highly rough
Hydrothermal^22^	70 ~ 212, tunable	60–120	392 nm (broad)	Width (by citrate/[Ag^+^] ratio)	Product morphology highly sensitive to [HCl]
Reduction on MAC (this work)	Mean 45 ~ 105, tunable	ca. 13	Two peaks at 335, 474 ~ 714 (tunable) nm	Width (by temperature)	Edges of nanobelts quite rough

**Table 2:** Comparison of five silver nanobelt samples obtained at different temperatures on their sizes (mean width/mean thickness) and visible light absorption peak wavelength.

Sample ID	#1	#2	#3	#4	#5
Preparation temperature (°C)	30	24	24	20	19
Mean width (nm)	105.4	67.5	62.4	61.8	45.3
Mean thickness (nm)	14.3	15.8	15.6	11.9	11.1
Width/Thickness	7.35	4.27	4.01	5.21	4.08
Visible peak wavelength (nm)	714	577	514	623	547
colour	light-blue	blue-purple	red	blue	purple
