# Supplementary material for: Tunable growth of silver nanobelts on monolithic activated carbon with size-dependent plasmonic response
Source: Sci Rep. 2015 Sep 4;5:13587. doi: 10.1038/srep13587 (PMC4559802; doi:10.1038/srep13587)
Supplement: Supplementary Information [file srep13587-s1.doc]

Supplementary Information

**Tunable growth of silver nanobelts on monolithic activated carbon with size-dependant plasmonic response**

Hong Zhao^1,3^, Yuesheng Ning^2,4^, Binyuan Zhao^2,4*^, Fujun Yin^1^, Cuiling Du^2^, Fei Wang^2^, Yijian Lai^2,4^, Junwei Zheng^3*^, Shuan Li^1^ & Li Chen^1^

1 Department of Chemical Engineering, Huaihai Institute of Technology, Jiangsu Marine Resources Development Research Institute, Lianyungang 222005, P. R. China.

2 State Key Laboratory of Metal Matrix Composites, School of Materials Science and Engineering, Shanghai Jiao Tong University, Shanghai 200240, P. R. China.

3 College of Chemistry, Chemical Engineering and Materials Science, Soochow University, Suzhou 215123, P. R. China

4 Green Support Materials Technologies (Shanghai) Co. Ltd. Shanghai 200240, P.R. China

H.Z. and Y.N. contributed equally to this work.

*Correspondence and requests for materials should be addressed to B.Z. (byzhao@sjtu.edu.cn) or J.Z.(jwzheng@suda.edu.cn).


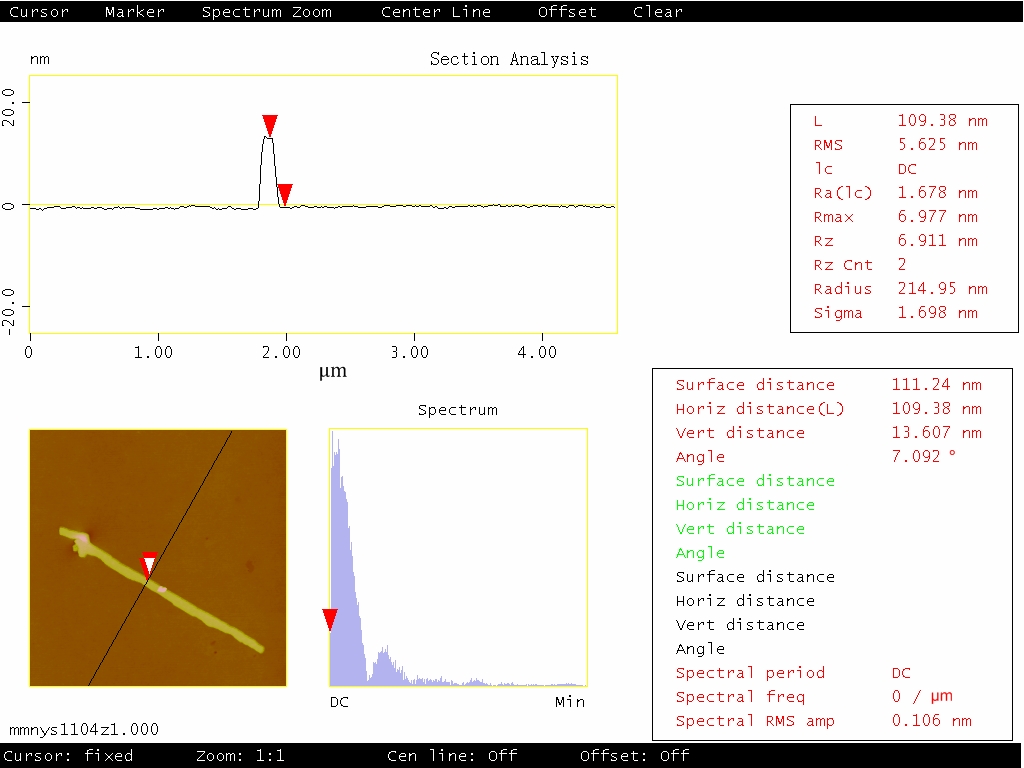


**Supplementary Figure S1 ︱A typical tapping mode AFM image of a silver nanobelt with section analysis. The red marker yields a vertical distance (i.e. thickness of the nanobelt) of 13.607 nm.**

**
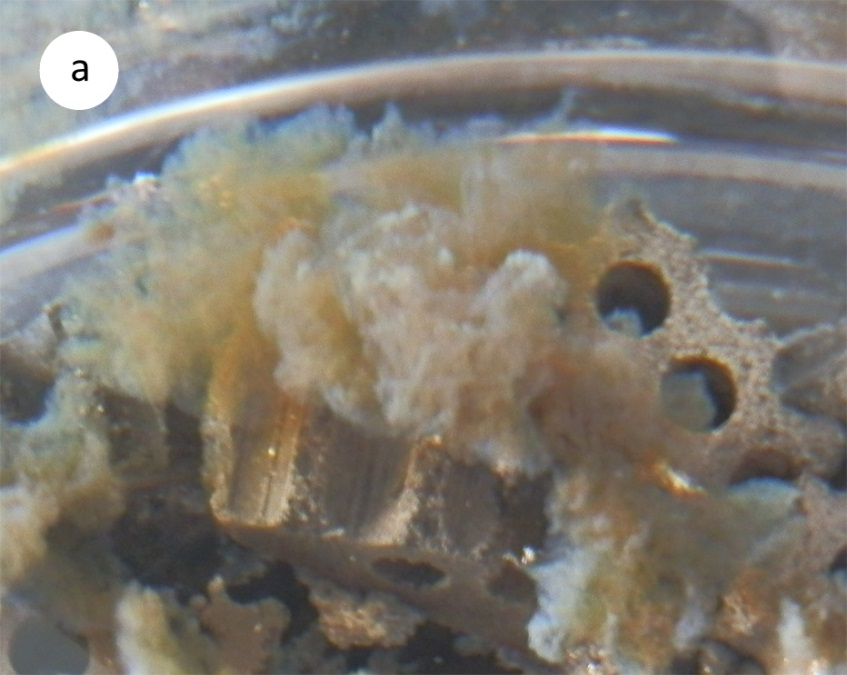
**

**Supplementary Figure S2 ︱Yellow silver nanobelts grown on MAC.** (a) Digital Photograph, (b) extinction spectrum corresponding to the yellow silver nanobelts in (a).

**Supplementary Figure S3 ︱XRD analysis of silver products.** (a) XRD pattern of five silver samples prepared on MAC, (b)(200)/(111) peak height ratio corresponding to (a). The (I) plate and (II) micro-belt samples were prepared as described in our previous work^1^. Samples (III), (IV) and (V) have the same color appearance as samples #2, #5 and #3, respectively, as shown in Figure 3.

XRD spectra of five silver samples prepared on MAC are presented in Supplementary Fig. S3a. All of them have five clean peaks and can be indexed to the (111), (200), (220), (311) and (222) characteristic planes of face centered cubic (fcc) silver, which match well with the standard pattern of silver (JCPDS, NO. 04-0783). According to a structural model depicted by Wang^2,3^ , the basal surfaces of Ag nanobelts, triangle nanoprisms and hexagonal nanoplates are {111} planes, and each of their side surfaces consists of two {100} planes joined by a stacking fault ridge (or groove). So the {111}/{100} height ratio should be positively correlated to the width/thickness ratio. The (200)/(111) peak height ratio (inverse to the above-mentioned {111}/{100} ratio) derived from Supplementary Fig. 3a is plotted in Supplementary Fig. S3b. The red, purple and blue-purple nanobelts, which possess consecutively higher width/thickness ratio as listed in Table 1, exhibit lower (200)/(111) height ratio in order. This result further confirms that the red-shift in transversal plasmon peaks in the extinction spectra is actually caused by the increase in the width/thickness ratio^4^.

**Supplementary References**

1. Zhao, H. *et al.* “Green “planting” nanostructured single crystal silver”. *Sci. Rep.* *3*, 1511, (2013) .
2. Wang, Z.L. Transmission Electron Microscopy of Shape-Controlled Nanocrystals and Their Assemblies, *J. Phys. Chem. B* 104, 1153-1175, (2000).
3. Le Beulze, A. et al. New Insights into the Side-Face Structure, Growth Aspects, and Reactivity of Ag_n_ Nanoprisms, *Langmuir* 30, 1424-1434 (2014).
4. Anderson, L.J. E., Payne, C.M., Zhen, Y.R., Nordlander, P., Hafner, J.H. A Tunable Plasmon Resonance in Gold Nanobelts, *Nano Lett.* 11, 5034–5037 (2011).
